# Attraction of nocturnal scarab beetles by unusual floral volatiles in a *Banksia* (Proteaceae) with functionally diverse pollinators

**DOI:** 10.1111/plb.70226

**Published:** 2026-06-02

**Authors:** S. K. Wawrzyczek, B. Bohman, S. L. Krauss, I. M. Butler, G. R. Flematti, K. Farnier, S. E. Hoebee, R. A. Davis, R. D. Phillips

**Affiliations:** ^1^ Department of Ecological, Animal and Plant Sciences La Trobe University Bundoora Victoria Australia; ^2^ School of Molecular Sciences The University of Western Australia Crawley Western Australia Australia; ^3^ Kings Park Science, Department of Biodiversity, Conservation and Attractions Kings Park Western Australia Australia; ^4^ Department of Plant Protection Biology Swedish University of Agricultural Sciences Lomma Sweden; ^5^ School of Biological Sciences The University of Western Australia Crawley Western Australia Australia; ^6^ Central Analytical Research Facility Queensland University of Technology Brisbane Queensland Australia; ^7^ School of Science Edith Cowan University Joondalup Western Australia Australia; ^8^ Royal Botanic Gardens Victoria South Yarra Victoria Australia

**Keywords:** cantharophily, chemical attractants, pollinator functional groups, SPME, vane traps, vertebrate pollinators

## Abstract

- Pollination by beetles has evolved multiple times in flowering plants but with relatively few plant species adapted specifically to pollination by scarab beetles (Coleoptera: Scarabaeidae). However, some plant species may produce floral scents and offer floral rewards that attract scarabs alongside other pollinator functional groups. In *Banksia attenuata* (Proteaceae), nocturnal observations led to the discovery of extensive floral visitation by beetles, alongside birds, mammals and diurnal insects. Given the distinctive melon‐like floral scent of *B. attenuata*, we predicted long‐distance attraction of beetles by chemical cues.
- We undertook floral visitor surveys aided by camera traps and video recordings, and quantified insect pollen loads. We used GC–MS to identify candidate beetle‐attracting compounds, which we then synthesised and tested in field bioassays.
- Honeyeaters, Honey possums and diurnal insects visited the flowers of *B. attenuata*. However, the most frequent visitors were nocturnal scarab beetles: *Pachytricha minor* and *Phyllotocus occidentalis* (Scarabeidae: Melolonthinae). Both beetle species fed on nectar and pollen and mated on the flowers. Beetles caught in the bioassays carried pure *B. attenuata* pollen. Floral volatiles of *B. attenuata* were dominated by two unusual compounds: 3,6‐nondien‐1‐yl acetate and 3,6‐nondien‐1‐ol that were not detected in 22 congeners. A synthetic mixture of these compounds proved strongly attractive to *Phyllotocus occidentalis* in the field.
- Nocturnal scarab beetles are a prominent component of the functionally diverse pollinators of *B. attenuata*. The attraction of *Phyllotocus occidentalis* by unusual floral volatiles suggests a case of adaptation to pollination by beetles among Australian Proteaceae, with remarkable parallels to some other plants pollinated by scarabs.

## Graphical abstract

*Banksia attenuata* (Proteaceae) attracts nocturnal scarab beetles with an unusual floral scent—the beetles pollinate the flower as they feed on pollen and nectar and mate on the inflorescences.

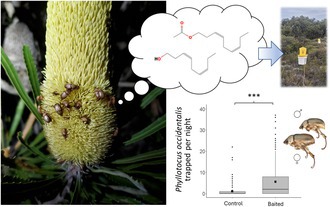

## INTRODUCTION

In flowering plants, pollination syndromes are sets of floral traits that have evolved repeatedly in response to selection from different pollinator functional groups (Willmer 2011; Ashworth *et al*. 2015). However, distinct variants of the major pollination syndromes continue to be described (Dellinger 2020). One of the most varied pollination systems in terms of floral traits is cantharophily, or pollination by beetles. The multitude of floral characteristics considered adaptations to pollination by beetles is likely a consequence of the extreme diversity of Coleoptera and the many ways that they can interact with flowers (Bouchard *et al*. 2017). For example, plants pollinated by beetles with coprophagous larvae may attract the adult beetles through mimicry of oviposition sites (Punekar & Kumaran 2010; Sayers *et al*. 2020), while other beetle‐pollinated plants may provide food rewards, including pollen, nectar and/or floral tissues (Beac 1982; Gibernau *et al*. 1999; Barfod *et al*. 2011). Alternatively, floral thermogenesis and provision of protected mating sites also feature prominently among cantharophilous plants (Procheş & Johnson 2009; Maia *et al*. 2010; Milet‐Pinheiro *et al*. 2017; Favaris *et al*. 2020). The diversity of traits potentially involved in attraction of beetle pollinators, in combination with their often‐nocturnal activity, raises the possibility that pollination by beetles may be more widespread than is currently recognised (Bernhardt 2000; Muinde & Katumo 2024).

Pollination by scarab beetles (Coleoptera: Scarabaeidae) is known from few plant lineages, but is a prominent feature of several plant families, particularly in the Neotropics and South Africa (Bernhardt 2000). These plants form a spectrum from pollination generalisation, where generalist flower‐visiting beetles contribute to pollination alongside other pollinators (Mayer *et al*. 2006; Johnson *et al*. 2007; Steenhuisen & Johnson 2012), to pollination specialisation, where beetles rely strongly on the flowers of a particular species and are its primary pollinators (Beach 1982; Ollerton *et al*. 2003; Maia *et al*. 2010; Favaris *et al*. 2020; Pinheiro‐Costa *et al*. 2025). The latter typically involves floral traits enhancing effective pollination by beetles, including emission of attractive floral volatiles and specialised food rewards. Such traits feature prominently in several lineages of Araceae and Annonanceae (Young 1986; Maia *et al*. 2010, 2012, 2013; Costa *et al*. 2017; Milet‐Pinheiro *et al*. 2017; Sayers *et al*. 2020), although they are also known from other plants that are pollinated by scarab beetles as part of a more generalised pollination system (Beach 1982; Ollerton *et al*. 2003; Johnson *et al*. 2007; Steenhuisen *et al*. 2012, 2013; Steenhuisen & Johnson 2012; Favaris *et al*. 2020).

As in other parts of the world, in Australia, many beetles visit flowers for food and to find mates. For some plants, they comprise a large portion of the floral visitors, which suggests they could make an important contribution to pollination (Phillips *et al*. 2015; Scaccabarozzi *et al*. 2020; Burns 2024). However, few Australian studies have documented targeted attraction of pollinating beetles to flowers. The systems that have been described involve either the honest advertisement of safe mating sites and food rewards for the brood (Armstrong & Irvine 1990) or mimicry of oviposition sites (Sayers *et al*. 2020). Yet there are large gaps in knowledge of the pollination ecology of many plant lineages where pollination by beetles could be expected based on floral traits.

One example is the Gondwanan plant family Proteaceae, where pollination by beetles (Scarabaeidae: Cetoniinae) occurs in several species of *Protea* L. (Steenhuisen & Johnson 2012), a South African genus that is superficially similar to the Australian genus *Banksia* L.f. (Collins & Rebelo 1987). In both genera, most species studied to date are primarily pollinated by birds and/or mammals (Hopper 1980; Paton & Turner 1985; Cunningham 1991; Carthew 1993; Wooller & Wooller 2003; Wawrzyczek *et al*. 2024, 2025a) typically with a minor contribution by a range of insects (Paton & Turner 1985; Ramsey 1988; Wawrzyczek *et al*. 2025a). So far, the only evidence of pollination by beetles in Australian Proteaceae has been provided by Lamont (1982), who observed scarab beetles of the genus *Pachytricha* Sharp, 1874 (Scarabaeidae: Melolonthinae) foraging on the flowers of *Grevillea leucopteris* Meisn. in a way consistent with effective pollination, although these observations were made in a garden setting outside of this species' natural geographic range.

Of the approximately 180 species of *Banksia*, the Western Australian *Banksia attenuata* R.Br. is remarkable because of its unusual floral traits: oily pollen (Ladd *et al*. 1996), short styles (Wiens *et al*. 1979), and strong fruity scent. Past studies using observational data and pollen loads suggested that *B. attenuata* is pollinated by birds (Whelan & Burbidge 1980; Frick *et al*. 2014; Ritchie *et al*. 2021) and non‐flying mammals, particularly the marsupial Honey possum (*Tarsipes rostratus*, Tarsipedidae) (Wiens *et al*. 1979; Hopper 1980). However, birds visit the flowers of *B. attenuata* much less frequently and carry less of its pollen compared to other co‐occurring *Banksia* species (Wiens *et al*. 1979; Hopper 1980; Whelan & Burbidge 1980; Ritchie *et al*. 2021). Moreover, observations of insects visiting the flowers (Lewis & Bell 1981), along with studies employing selective exclusion of vertebrate pollinators, indicated that invertebrates can be effective pollinators of this species (Whelan & Burbidge 1980; Wooller & Wooller 2001).

The present study was conceived following the unexpected discovery of nocturnal visitation of *B. attenuata* flowers by scarab beetles. Preliminary surveys conducted in a *Banksia* woodland revealed that at night considerable numbers of the large (25 mm‐long) *Pachytricha minor* (Melolonthinae) visited the flowers alongside other smaller (8 mm‐long) but more numerous *Phyllotocus occidentalis* (Melolonthinae). These surveys also revealed that at the same site, floral visitation by birds and mammals was very infrequent, while the only insect observed visiting the flowers during the day was the introduced European honeybee (*Apis mellifera*). These observations led us to hypothesise that the beetles could be overlooked but important pollinators of *B. attenuata* and that they are attracted to the flowers by the unusual floral scent of this species. To test these hypotheses, we quantified floral visitation by potential pollinators of *B. attenuata* at six sites, quantified pollen loads on insects and compared the foraging behaviour of beetles and other potential pollinators. Further, following GC–MS‐based analyses of the floral volatiles, we synthesised two candidate beetle‐attracting compounds and conducted a field bioassay to test whether these specific compounds were attractive to the beetles.

## METHODS

### Study species and sites

**Fig. 1:**
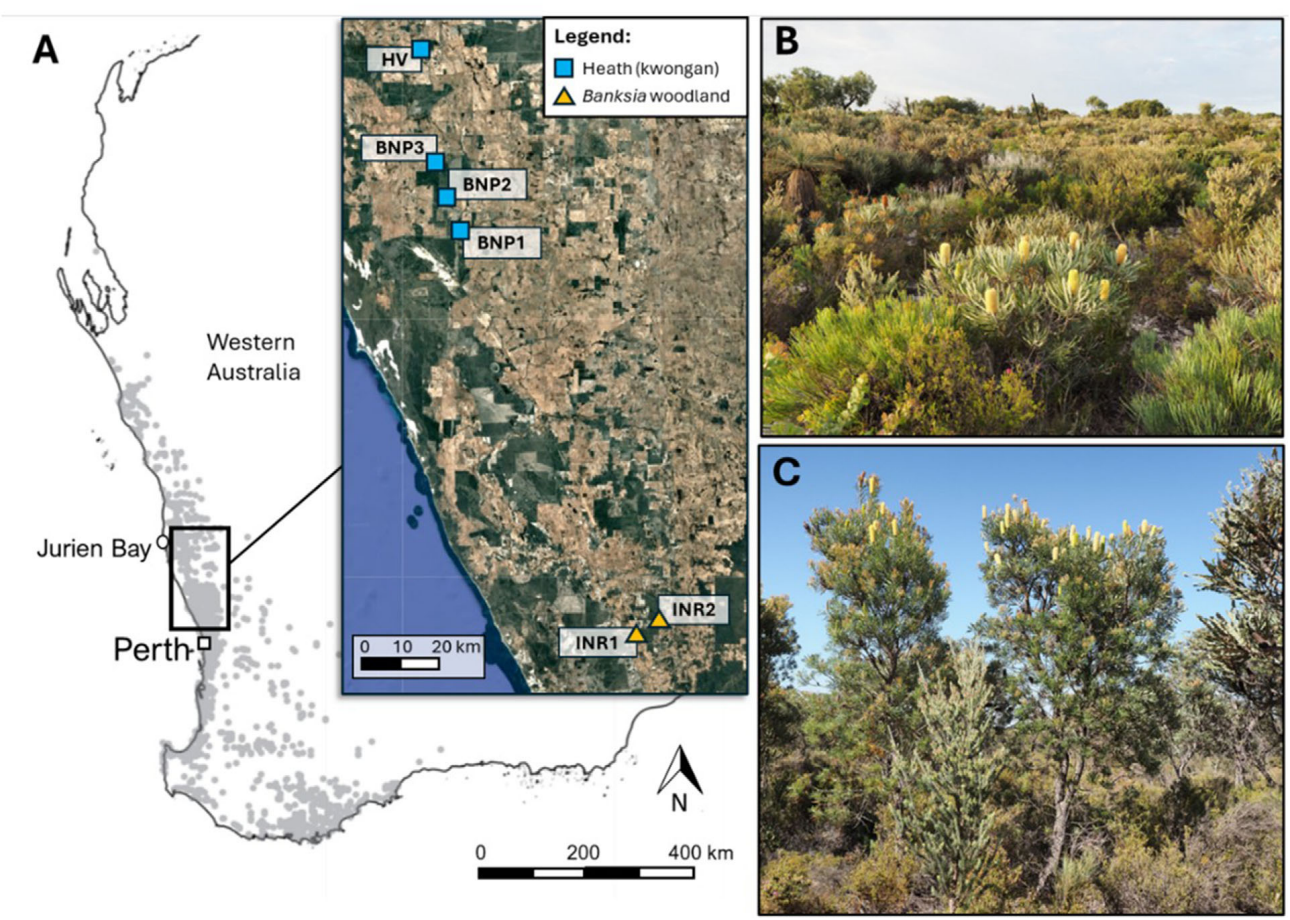
(A) Map of southwest Western Australia showing records of occurrence of *Banksia attenuata* (grey dots, Atlas of Living Australia) and location of study sites at Hi Vallee Farm (HV), Badgingarra National Park (BNP) and Ioppolo Nature Reserve (INR); (B) shrub‐form *B. attenuata* in a heathland at BNP2; (C) tree‐form *B. attenuata* in a *Banksia* woodland at INR1.

*Banksia attenuata* R.Br. (Proteaceae) is a tree or shrub with a wide distribution in southwest Western Australia (Fig. 1). It grows as a tree to >10 m tall in higher rainfall parts of its geographic range, while in lower rainfall areas it typically grows as a stunted tree or shrub 1–4 m tall (George *et al*. 2021). Flowering from October to February, *B. attenuata* produces inflorescences of bright yellow flowers that are perceived by humans as strongly fruity‐scented (both during the day and night), reminiscent of melon or cucumber. As is common in Proteaceae, the individual florets comprising the inflorescence are protandrous with pollen secondarily presented at the tip of a gently curved style (15–22 mm long in *B. attenuata*, Ladd & Bowen 2020) where it initially covers the stigma (male phase of anthesis). Unusually for *Banksia*, the pollen is oily and suspended in viscous pollenkitt (Ladd *et al*. 1996). Several days after floret opening, the stigmatic groove opens and the flowers become receptive to pollen (female phase). The florets open continuously during the day and night, with the entire inflorescence completing anthesis over 7–15 days (Ladd *et al*. 1996).

Fruit set in *B. attenuata* is expected to depend on outcross pollination by animal pollen vectors (Scott 1980; Wooller & Wooller 2001). The fruits are large woody follicles, each containing up to two seeds. They are grouped in dense cone‐shaped infructescences and are weakly serotinous. In the first year of the study, a selective pollinator exclusion experiment was conducted at INR1. This experiment confirmed that in the absence of animal pollinators, *B. attenuata* did not set fruit (n = 6 inflorescences), and that small insects can be effective pollinators of this species (n = 28 inflorescences; see supporting information S1 for full details).

This study was conducted between 2020 and 2024 and focused on six sites up to 150 km apart, representative of open *Banksia* woodland and kwongan heath plant communities: Ioppolo Nature Reserve (INR1: 31° 29′ 53″S, 115° 57′ 55″ E; INR2: 31°28′29.59″S, 115°59′25.36″ E), Badgingarra National Park (BNP1: 30° 33′ 23”S, 115° 27′ 14″ E, BNP2: 30° 29′ 03”S, 115° 25′ 34″ E and BNP3: 30° 23′ 56.29″S, 115° 24′ 35.91″E) and Don & Joy Williams Nature Reserve at Hi Vallee Farm (HV: 30° 07′ 06″S, 115° 23′ 48″ E; Fig. 1). At each site over 100 flowering *B. attenuata* plants occurred within large (>400 ha) patches of long‐unburnt native vegetation. Herbarium vouchers for each reserve were lodged with La Trobe University Herbarium (LTB, catalogue numbers: 8022, 8025, 8148, 8648).

### Surveys of potential pollinators

To identify and quantify floral visitors and document their behaviour on the flowers, direct observations aided by video recordings and camera trapping surveys were undertaken from 2020 to 2024 between late November to early January. During direct observation surveys the researcher walked slowly through the site for 60 min in a pre‐determined direction, covering an area between 1,000 and 25,000 m^2^ depending on the size of the *B. attenuata* stand and flowering intensity. The researcher stopped every 5–10 min to note any birds or mammals visiting the flowers from a distance of 15–20 m, subsequently approaching the plants to score visitation by insects and observe their foraging behaviour at closer range. These surveys were conducted during the day between 06:00 h and 17:00 h and at night with a spotlight between 20:00 h and 23:00 h. Diurnal surveys were supplemented by video recordings, where one or two compact zoom cameras (Canon sx740) were set up on tripods focused on a single inflorescence or a tight cluster of up to 7 inflorescences and left unattended to record 30‐min videos (60 frames per second at FHD resolution).

Alongside these surveys, remote camera traps (Reconyx HC600 and HF2X) were used to monitor diurnal and nocturnal visitation to inflorescences. The cameras were modified by the manufacturer to focus at 1 m. They were either positioned on steel stakes up to 1.6 m off the ground or attached to the higher branches up to 6 m above the ground using steel L‐brackets and ball‐head clamp mounts. The cameras were set to very high sensitivity to maximise detection of small animals. When triggered, the cameras recorded a series of five photos followed by a 10 s video and a 5 s quiet period.

In total, we conducted 67.5 h of direct observation surveys during which we inspected 1960 inflorescences, supplemented by 29 h of video recordings of a total of 106 inflorescences. In parallel, the remote camera traps provided another 1921 h of surveys of floral visitation to a total of 62 inflorescences. The survey effort varied between the different pollinator survey methods, and overall, there was some bias toward daytime observations/recordings (i.e. from 05:00 h to 19:00 h; see Table S1 for full details).

### Pollen loads from beetles and other invertebrates

During the floral visitation surveys, individual insects were collected directly into the storage vials from *B. attenuata* inflorescences and from vane traps set among the flowering *B. attenuata* plants (details below). To determine whether they were capable of transporting *B. attenuata* pollen between the plants, all insects were inspected under a dissecting microscope and pollen samples were collected by rubbing the insects' entire bodies with a block of fuchsin‐stained gelatine (Wooller *et al*. 1983a). In the case of *Apis mellifera*, we avoided sampling pollen stored in the corbiculae considering it unlikely to be transferred to the stigmas. The gelatine was then transferred to a glass microscope slide, melted under a cover slip, and observed under a bright‐field microscope (Nikon Eclipse NiU, Tokyo, Japan). Pollen was also collected directly from freshly opened flowers of *B. attenuata* and co‐flowering species for identification. To allow a semiquantitative comparison of *B. attenuata* pollen loads among insect species, we categorised the loads based on the number of pollen grains detected in the samples as light (1–10 pollen grains), moderate (11–50 grains), heavy (51–100 grains) and very heavy (>100 grains). Because earlier studies detected *B. attenuata* pollen on birds (Wooller *et al*. 1983b) and small mammals (Saffer 1998), we did not repeat this work.

### Floral volatile sampling and chemical analyses

Floral volatiles were sampled overnight in a laboratory from the headspace of five inflorescences using Solid Phase Microextraction (SPME), with controls taken from leaves without flowers and from the ambient air. In addition, floral volatiles were sampled from two inflorescences through dynamic headspace sampling with Tenax® filters to allow determination of key compounds by co‐injection with authentic standards (see supporting information S1 for full details). The samples were analysed using gas chromatography–mass spectrometry (GC–MS), with automated deconvolution of peaks using AMDIS version 2.7. All compounds that were detected above an abundance threshold of 1000 (clearly visible, distinct peaks) were tentatively identified by comparing mass spectra (ion profiles) and retention indices with the NIST20 mass spectral library using NIST MS Search (version 2.3, NIST, USA). The three most abundant compounds that were consistently detected through both SPME and dynamic headspace sampling were identified by co‐injection with reference compounds (see supporting information S1 for full details).

### Scent attractiveness to beetles (vane trapping)

The bioassay was focused on nocturnal beetles as they were by far the most abundant native insects visiting *B. attenuata* flowers. Based on our analyses of the floral scent of *B. attenuata*, we proposed (3*Z*,6*Z*)‐nonadien‐1‐ol and (3*Z*,6*Z*)‐nonadien‐1‐yl acetate as potential beetle‐attracting volatiles. These two, closely related compounds consistently comprised a large proportion of the floral scent blend of *B. attenuata* and were absent from the leaves. Neither of these compounds was detected in 22 other *Banksia* species that we sampled using comparable methods (Wawrzyczek *et al*. 2026 and unpublished data), suggesting they could mediate targeted attraction of beetles in *B. attenuata* (Goodrich & Jürgens 2018).

For the field bioassay, 20 custom vane traps were built based on the design of a Japanese beetle trap but large enough for *P. minor* beetles to pass through the funnel opening. Each trap comprised a 2 L white plastic tub (17 cm diameter) fitted with a funnel (10 cm internal diameter, attached to the rim of the tub with wire clips) and 25 cm tall cross vane panels (interlocking with the funnel part). The funnel and cross vanes were made from rigid yellow art and craft card (A3 300gsm Colour Card ‘Lemon’, Liviano; Fig. S1).

Half of the traps in each array were baited with the synthetic lure comprising equal amounts of (3*Z*,6*Z*)‐nonadien‐1‐ol and (3*Z*,6*Z*)‐nonadien‐1‐yl acetate (at a concentration of 0.5 mg of each compound per 1 mL of hexane; see supplementary information for details of synthesis). This mixture was selected because these two compounds always occurred together in the flowers, in comparable relative abundance. The lure was presented in 4 mL screw top glass vials with 2 mm wide holes drilled in the lids and fitted with cotton wicks threaded through 2 mm wide Teflon tubing cut to 5 cm long sections (Birgersson & Lejfalk 1997). Comparisons of the total ion current in samples collected using SPME over 3 h indicated that at room temperature, at the concentration specified above, the amount of lure emitted from the wicks was comparable to one freshly cut inflorescence of *B. attenuata*. In the field, the vials containing the lure were attached to the side of the cross vanes below a central opening using Blu Tack (Bostick, Fig. S1). Paired negative controls were fitted with vials containing hexane only. In the field, the traps were set on bamboo stakes 1.2 m above the ground, in five regular arrays of four traps, with two baited and two control traps placed on diagonally opposite vertices of a square with a side of 5 m. The arrays were positioned among flowering plants of *B. attenuata*, at 5–10 m away from the nearest inflorescence and at least 5 m away from each other. (Fig. S1). Trapping was undertaken from 19:30 h to 23:30 h over 12 nights: 27–28‐Nov‐2023 at HV, 2–6‐Dec‐2023 concurrently at BNP1 and BNP2, and 5–6‐Dec2024 and 16–17‐Dec‐2024 at BNP3, which amounted to a total of 194 paired (i.e. baited + control) trap nights. The weather conditions during trapping nights were mostly clear (some nights were moon‐lit), with light or no wind, and temperatures ranging from 17°C to 22°C. Under these conditions, hexane evaporated from the vials at a rate of approximately 0.25 mL hr^−1^.

### Statistical analysis

To compare the rates of capture of beetles between baited and control traps, we used ‘glmmTMB’ package (Brooks *et al*. 2017) in R version 4.2.3 (R Core Team 2021) to fit a generalised linear mixed model with a negative binomial distribution (nbinom2) with a log‐link function. We specified trapping site nested in trapping date as random effects in the model.

## RESULTS

### Floral visitors

Direct observation contributed most of the observations of invertebrates (2555 in total), with occasional (17) vertebrates also observed directly. The surveys revealed considerable spatial variation in the visitor assemblages to *B. attenuata* flowers, with clear differences between the *Banksia* woodland (INR sites) and heathland sites (BNP and HV sites, Table 1).

**Table 1:** Floral visitation to *Banksia attenuata* by potential pollinators based on direct observation, video recordings and remote camera traps. Values are numbers of visits to individual inflorescences, pooling data from all surveys (*N*). D = day (05:00–19:00 h), N = night (19:00–05:00 h). Icons indicate functional groups of potential pollinators: birds, specifically honeyeaters (Meliphagidae), non‐flying mammals, lizards, beetles (Coleoptera), moths (Lepidoptera), flies (Diptera), bees and wasps (Hymenoptera).

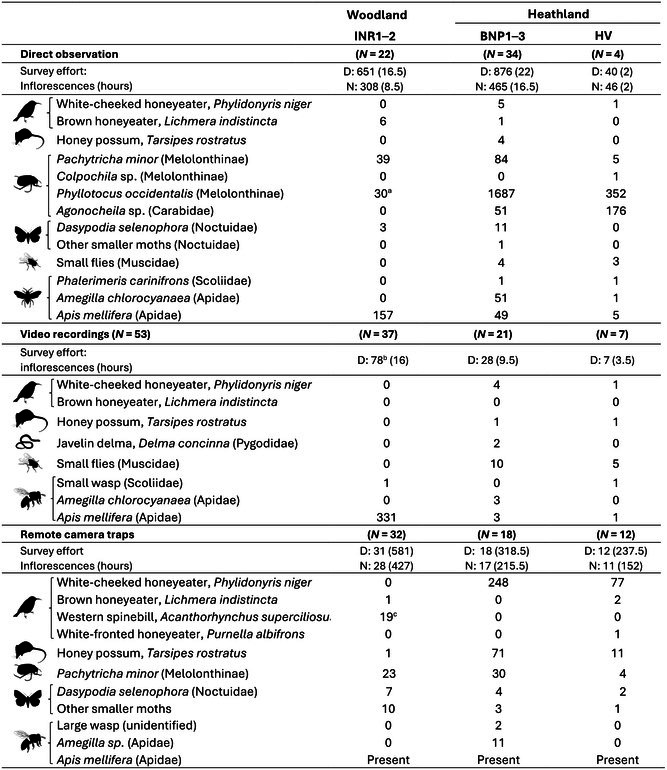

^a^ Thirty individuals were found buried among the florets of two inflorescences that were collected for herbarium vouchers in January 2021. Otherwise, these beetles were too small to quantify because most inflorescences at INR sites were several meters above the ground. In January 2022 large number of small beetles were incidentally recorded by one of the camera traps. However, in November 2023 and early December 2024 several inflorescences lower to the ground were inspected and no beetles were detected.

^b^ In several surveys there were multiple (up to six) inflorescences in the field of view of the camera.

^c^ All visits recorded by a single camera early in the flowering season.

Combining all survey methods, four bird species (Meliphagidae) were observed visiting inflorescences of *B. attenuata*: the White‐cheeked honeyeater (*Phylidonyris niger*, Bechstein, 1811), Brown honeyeater (*Lichmera indistincta*, Vigors & Horsfield, 1827), Western spinebill (*Acanthorhynchus superciliosus*, Gould, 1837) and White‐fronted honeyeater (*Purnella albifrons*, Gould, 1841). Most bird visits were by White‐cheeked honeyeaters (336, 92%) at the heathland sites. The cameras recorded only sporadic visits by Brown and White‐fronted honeyeaters, with 19 visits by Western spinebills recorded on a single inflorescence by one of the camera traps in the woodlands. Honey possums were the only mammals detected, with 82 visits recorded in the heathlands and a single visit recorded in the woodlands (Table 1). The video recordings also captured two floral visits by Javelin lizards (*Delma concinna*, Pygopodidae Kluge, 1974), a typically ground‐dwelling legless lizard (Table 1).

Overall, the most frequent invertebrate visitors were nocturnal beetles: the large *Pachytricha minor* Sharp, 1874 (c. 25 mm long), the smaller *Phyllotocus occidentalis* Blackburn, 1888 (Scarabaeidae: Melolonthinae) (c. 8 mm long) and a small ground beetle *Agonocheila* sp. (Carabidae: Harpalinae, c. 7 mm long) (Table 1). In total, 185 visits by *Pachytricha minor* were detected (combining direct observation and camera trapping), with a further 2069 visits by *Phyllotocus occidentalis* and 227 visits by *Agonocheila* sp. Counting only the visits that were observed directly, these three beetles accounted for 89% of all invertebrates, despite the slight bias toward daytime observations. Other nocturnal visitors occasionally comprised moths, including the large *Dasypodia selenophora* and other, smaller species of the family Noctuidae.

**Fig. 2:**
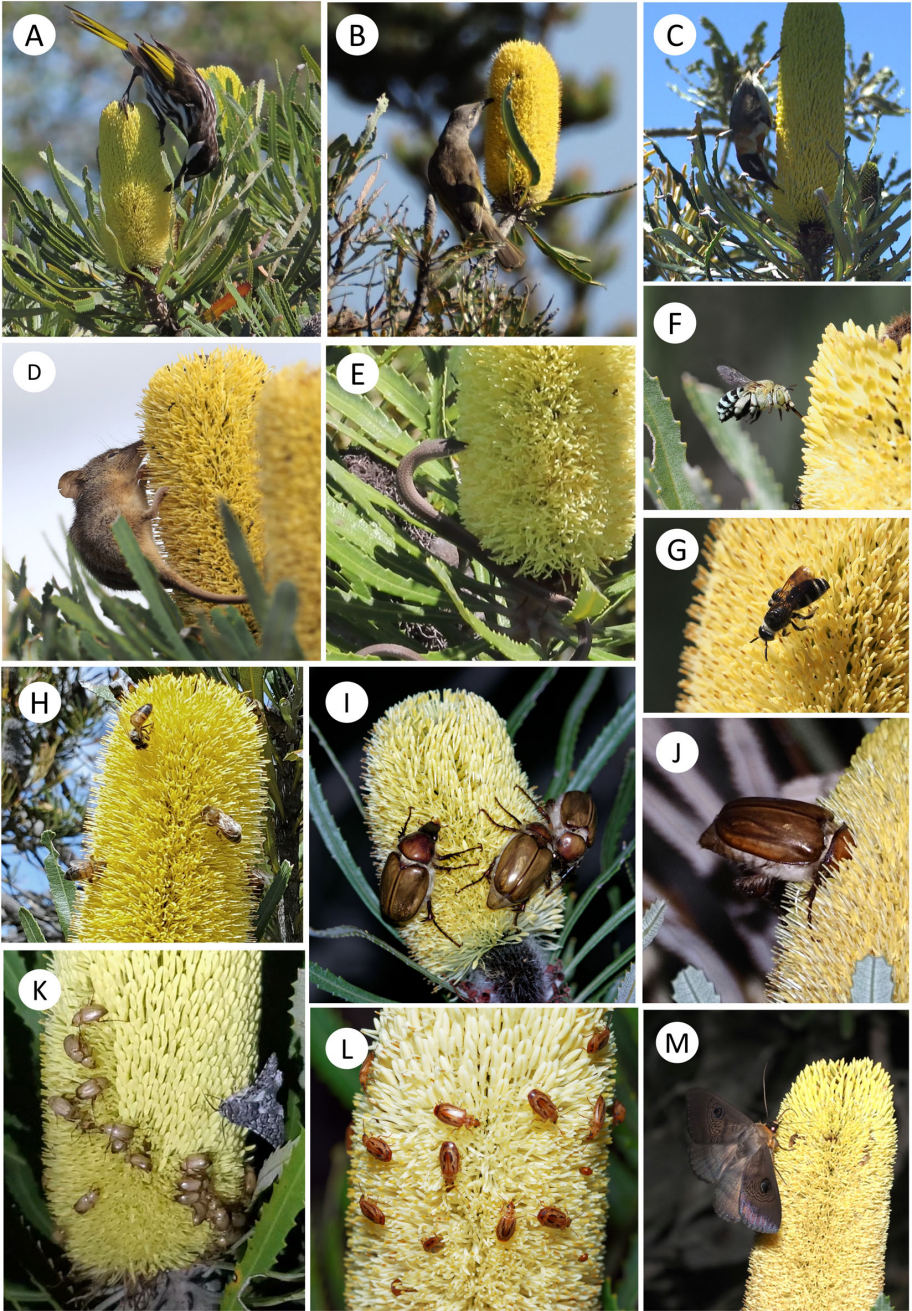
Potential pollinators of *Banksia attenuata*. (A) White‐cheeked honeyeater; (B) Brown honeyeater; (C) Western spinebill; (D) Honey possum; (E) Javelin delma; (F) *Amegilla chlorocyanaea*; (G) *Phalerimeris carinifrons*; (H) *Apis mellifera*; (I, J) *Pachytricha minor*; (K) *Phyllotocus occidentalis* and unidentified moth (Noctuidae); (L) *Agonocheila* sp.; (M) *Dasypodia selenophora*.

With regards to diurnal invertebrates, the native bee *Amegilla chlorocyanaea* (Apidae) was a frequent visitor to the flowers at one of the heathland sites (BNP2), but only sporadic elsewhere. Flies and wasps were recorded only sporadically (Fig. 2, Table 1). In addition, the introduced honeybee (*A. mellifera*) was observed visiting the flowers, particularly at the woodland site, where it was the most frequent visitor.

Ants, weevils and other very small beetles (<3 mm long), and occasionally katydids were also seen on the flowers but were not counted because they are unlikely to be effective pollinators of *Banksia* (they very rarely contacted pollen presenters and were not seen to move between inflorescences).

### Foraging behaviour of potential pollinators

The birds typically alighted directly on the inflorescences above the row of the most recently opened florets and repeatedly inserted their bills into the florets, often systematically moving down the flower spikes. In all cases the birds' bills appeared to touch the reproductive parts of many individual florets. However, because the stigma‐nectary distance in *B. attenuata* is considerably shorter than the bills of the White‐cheeked and Brown honeyeaters, little or no pollen would be transferred to the birds' feathers (Video S1). Honey possums appeared to forage mostly for nectar by repeatedly burying their faces deep among the florets. While they were foraging, large areas of their bodies were rubbing on the stigmas/pollen presenters below the advancing front of the most recently opened florets (Video S1). Honey possums were observed/recorded mostly at night but occasionally also during daylight (Fig. 2D). The two Javelin lizards recorded appeared to forage for nectar by inserting their heads among the florets, touching the stigmas/pollen presenters in the process (Video S1).

The beetles were observed visiting *B. attenuata* flowers at night, with the first beetles arriving soon after dark (ca. 20:00 h) and continuing to forage at least until midnight. *Pachytricha minor* tended to forage on individual inflorescences for several minutes without moving between inflorescences. However, because of their large size, during the nocturnal surveys they could be heard flying among the flowering *B. attenuata* plants and were recorded alighting on the inflorescences at various times through the night, with one *P. minor* recorded flying off the inflorescence without being disturbed. All *P. minor* fed on pollen without visibly damaging the flowers and occasionally buried their heads between the florets (Video S1). As they foraged, they crawled over the inflorescences with the hairy ventral surfaces of their bodies touching the stigmas/pollen presenters. *P. occidentalis* foraged in a similar way to *P. minor*, however, due to their smaller size it was not clear whether they ingested pollen. Both beetle species were frequently observed mating on the flowers (Video S1), with some individuals combining mating and feeding in the same visit.

*Dasypodia selenophora* (Noctuidae) moths were observed feeding on the flowers by inserting their proboscides between the florets. Otherwise, they rarely appeared to touch the reproductive parts of the flowers except with the legs and proboscis (Video S1). Smaller moths were more likely to touch the stigmas while foraging.

The bees foraged for nectar and/or pollen. The native bee *Amegilla chlorocyanaea* (Apidae) made frequent, short visits to the flowers. Individuals of the introduced honeybee *Apis mellifera* (Apidae) foraged either for pollen, mostly in the morning, or nectar, at any time of the day. Individual *A. mellifera* tended to remain on the inflorescences for several minutes before departing. Wasps were sporadic visitors of *B. attenuata*. *Phalerimeris sp*. (Scoliidae) was observed on two occasions briefly feeding on the flowers in a way similar to native bees. Muscid flies occasionally fed from the surface of the stigmas.

### Pollen loads

**Fig. 3:**
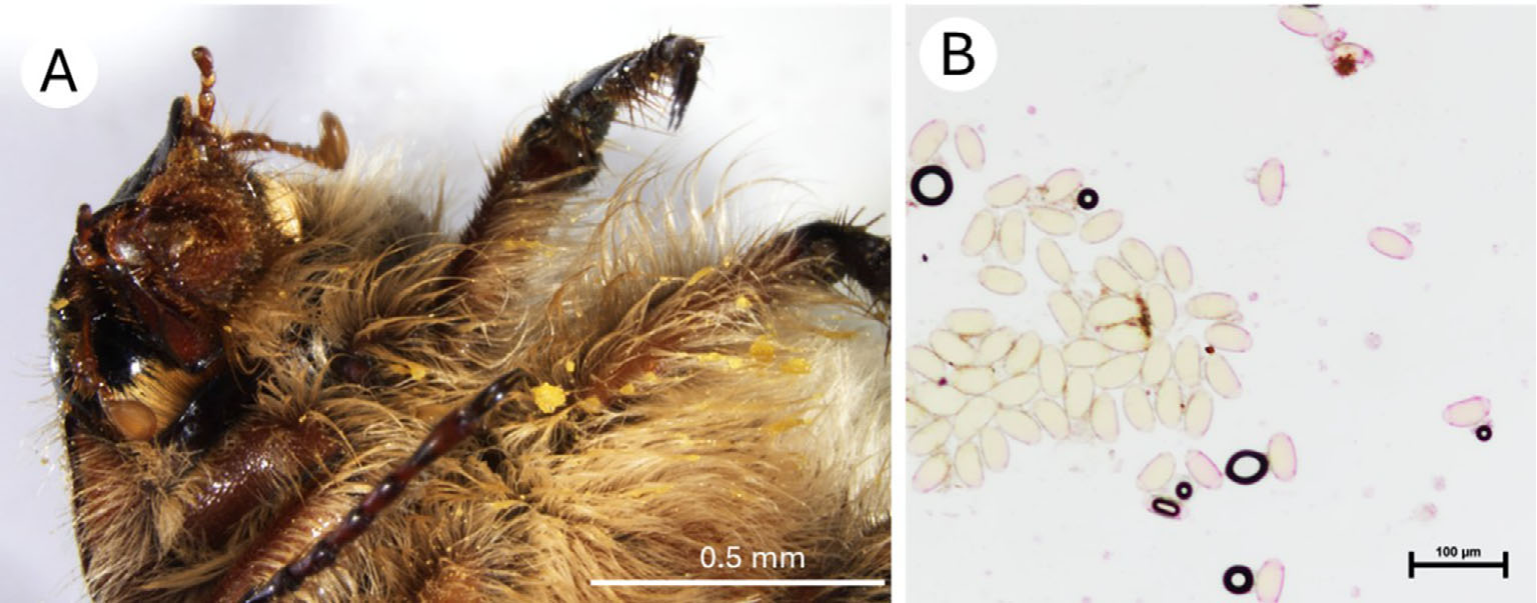
(A) *Pachytricha minor* beetle collected directly from an inflorescence of *Banksia attenuata* with lumps of yellow pollen clearly visible under dissecting microscope; (B) example of fuchsin‐stained pollen sample collected from *P. minor* caught in one of the vane traps. The size and shape of the pollen matched reference pollen samples taken directly from *B. attenuata* flowers.

*Pachytricha minor* beetles collected from *B. attenuata* flowers always carried either heavy (*N* = 3) or very heavy (N = 8) loads of pure *B. attenuata* pollen (Fig. 3), while those caught in vane traps carried at least moderate loads (N = 9). The single individual of the *Colpochila* sp. (Scarabaeidae: Melolonthinae) collected from the flower carried moderate loads. *Phyllotocus occidentalis* beetles collected from flowers carried light to moderate pollen loads (N = 3), while those caught in vane traps mostly carried light pollen loads (N = 14) or none (N = 4). Two individuals of the *Agonocheila* sp. (Carabidae: Harpalinae) collected from flowers carried moderate pollen loads.

Of the remaining insects that were collected from flowers, an unidentified small moth (Noctuidae) carried a light pollen load (N = 1), while no pollen was detected on *Dasypodia selenophora* (N = 2). *Amegilla chlorocyanaea* bees carried light loads of *B. attenuata* mixed with moderate or heavy loads of other species (N = 3). *Apis mellifera* carried very heavy loads of pure *B. attenuata* pollen (N = 3).

### Floral volatiles

The floral volatiles of *B. attenuata* were dominated by three compounds: (3*Z*,6*Z*)‐nonadien‐1‐ol, (3*Z*,6*Z*)‐nonadien‐1‐yl acetate and (*E*)‐β‐ocimene (Table S2, Fig. S2). However, (*E*)‐β‐ocimene was also detected as a major component of the leaf volatiles (Table S2, Fig. S2). The three compounds were also detected through dynamic headspace sampling and were confirmed with authentic standards.

Minor components of the floral odour were tentatively identified as: 2,6‐nonadienyl acetate, methyl salicylate, 3‐hexen‐1‐yl acetate, several monoterpenes (structural isomers of β‐ocimene), sesquiterpenes (α‐farnesene, gymnomitrone) and nerolidol, with additional compounds detected in very small amounts or only in one or two samples (Table S2). Apart from (*E*)‐β‐ocimene, the control samples collected from leaves contained mostly caryophyllene and α‐farnesene (Table S2).

### Scent attractiveness to beetles

**Fig. 4:**
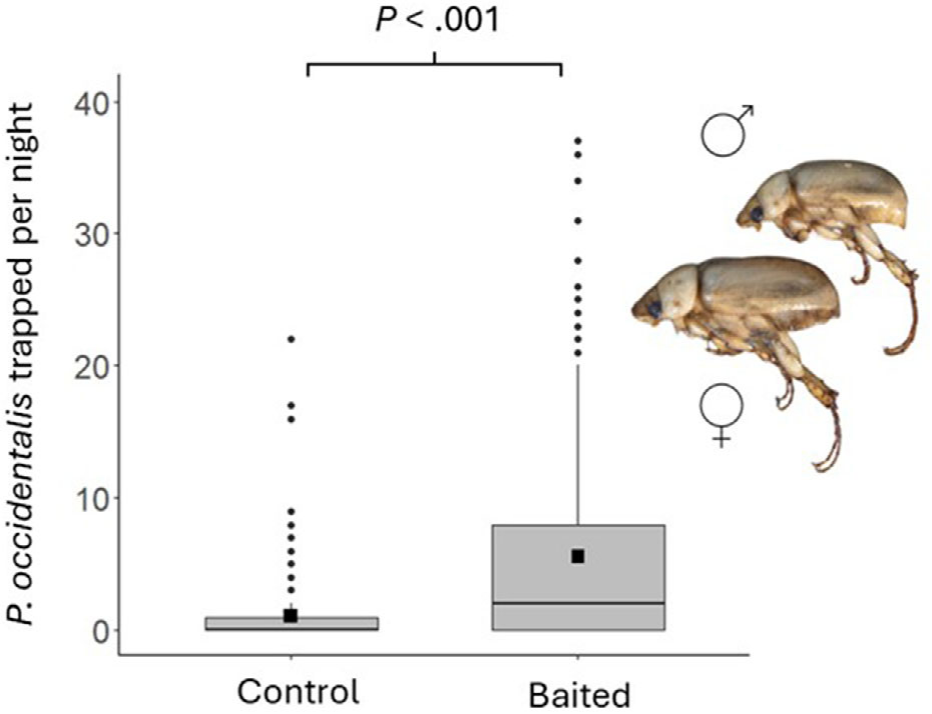
Comparison of the trapping rates of *Phyllotocus occidentalis* (male and female combined) between control and baited traps (N = 194 paired trap nights). Black squares indicate group means. *P*‐value based on GLMM fitting negative binomial distribution to the data. The image shows male and female *P. occidentalis*.

In the field trials, vane traps baited with the lure comprising a mixture of (3*Z*,6*Z*)‐nonadien‐1‐ol and (3*Z*,6*Z*)‐nonadien‐1‐yl acetate attracted significantly more *P. occidentalis* beetles than unbaited controls (1076 vs 204, respectively; GLMM: estimate = 2.09 ± 0.19, z = 10.9, *P* < 0.001; Fig. 4). The proportion of male and female beetles caught could not be determined because of the absence of obvious sexual dimorphism. However, both sexes were trapped, as indicated by many individuals seen mating in the traps. Six individuals of *Pachytricha minor* were caught in baited traps and two in control, which was insufficient for a statistical test. Only two individuals of other insects were caught: one *Colpochila* sp. and one moth (Noctuidae), both in control traps.

## DISCUSSION

Our results supported the hypothesis that nocturnal beetles may be important pollinators of *Banksia attenuata*—likely contributing substantially to pollination alongside a diversity of vertebrate and other invertebrate visitors. Both *Pachytricha minor* and the smaller but more abundant *Phyllotocus occidentalis* were very frequent floral visitors, with observations of foraging behaviour and pollen loads indicating that both species are capable of transferring large amounts of pollen. The potential role of *P. occidentalis* as a pollinator is consistent with its strong attraction to (3*Z*,6*Z*)‐nonadien‐1‐ol and (3*Z*,6*Z*)‐nonadien‐1‐yl acetate in the floral scent of *B. attenuata*. As anticipated based on past studies (Hopper 1980; Whelan & Burbidge 1980; Wooller & Wooller 2001), the potential pollinators of this species also included honeyeaters, the Honey possum and various diurnal insects. While we did not collect pollen load data for birds at our study site, high‐resolution videos of White‐cheeked honeyeaters feeding at the flowers suggest that birds may be relatively ineffective pollinators of this species due to a mismatch in floral morphology and beak length. In addition, vertebrates were infrequent visitors to the flowers at woodland sites, suggesting that some populations of *B. attenuata* may rely heavily on beetles for pollination.

The selective pollinator exclusion experiment confirmed that small insects can be effective pollinators of *B. attenuata*. However, the effectiveness of beetles as pollinators could not be determined because of the concurrent visitation of the flowers by introduced *A. mellifera*. Nonetheless, there are reasons that suggest both *Phyllotocus occidentalis* and *P. minor* are effective pollinators of *B. attenuata*. Although they consume pollen, they forage without damaging the flowers, in such a way that would result in some transfer of pollen to receptive stigmas. Crucially, both species were caught in vane traps carrying large, pure *B. attenuata* pollen loads, which indicates they can move pollen between the plants. An important area for future research would be to compare the contribution to fruit set and the genetic component of pollinator effectiveness of beetles and other pollen vectors for *B. attenuata* (Valverde *et al*. 2019; Wawrzyczek *et al*. 2025b).

Our study is one of very few to shed light on the contribution of unusual floral volatiles to the attraction of beetles to flowers. We showed that a mixture of two structurally related compounds, (3*Z*,6*Z*)‐nonadien‐1‐ol and (3*Z*,6*Z*)‐nonadien‐1‐yl acetate from the inflorescences of *B. attenuata* attract *P. occidentalis*. While too few individuals of *Pachytricha minor* were caught in the traps for a formal test, we suspect that they may use the same scent cues to find the flowers as *Phyllotocus occidentalis*. In *Banksia*, (3*Z*,6*Z*)‐nonadien‐1‐ol and (3*Z*,6*Z*)‐nonadien‐1‐yl acetate may be uniquely associated with *B. attenuata*, as these compounds were not detected in the flowers of 22 other *Banksia* sp. that were screened using comparable methods (Wawrzyczek *et al*. 2026 and unpublished data). In other plants, these compounds appear to be associated with fruits (particularly of the Cucurbitae, e.g. Wang & Lin 2014; Kourkoutas *et al*. 2006), rather than flowers (Knudsen *et al*. 2006). Notably, similar C9 compounds are used as components of the aggregation pheromones in several lineages of Scarabaeidae (e.g. Zilkowski *et al*. 2006; Serrano *et al*. 2019; Silva *et al*. 2021), which could suggest that they evolved in *B. attenuata* under selection to match the pre‐existing (and potentially ancient) sensory bias for similar compounds in the pollinating beetles (Schiestl & Dötterl 2012; Goodrich & Jürgens 2018; Koski 2020). The third main component of *B. attenuata* floral scent, (*E*)‐β‐ocimene, is a common plant volatile. It was consistently detected as the major component of the floral blends of all 22 other *Banksia* species that we sampled and was also present in the leaves (Wawrzyczek *et al*. 2026 and unpublished data).

To our knowledge, the only published study that tested the importance of floral scent in attracting *Phyllotocus* beetles is the work of Allsopp & Cherry (1991) from eastern Australia, who demonstrated attraction of *Phyllotocus navicularis* by several common floral volatiles, with the strongest attraction elicited by anethole (1‐methoxy‐4‐[(1*E*)‐prop‐1‐en‐1‐yl]benzene). The suggestion that one or two specific compounds from the flowers of *B. attenuata* are sufficient to attract *Phyllotocus occidentalis* aligns with the findings of several studies of plants that are ecologically specialised for pollination by scarabs. Such plants typically attract the pollinating beetles with a single rare compound or a simple mixture of a few compounds (Jürgens *et al*. 2000; Knudsen *et al*. 2001; Maia *et al*. 2012, 2013; Favaris *et al*. 2020). For example, 4‐methyl‐5‐vinylthiazole—a single sulfureous compound not previously reported from flowers—mediates the attraction of multiple species of pollinating beetles to the flowers of four *Anonna* species (Anonnaceae) (Maia *et al*. 2012). In *B. attenuata*, floral traits enhancing beetle pollination likely co‐exist with other traits not involved in the attraction/reward of beetles because of parallel selective pressures exerted by different pollinator functional groups (Aigner 2001; Pauw *et al*. 2020). For example, compared to certain Araceae and Annonaceae pollinated exclusively by beetles, the floral scent of *B. attenuata* is not as strongly dominated by only one or two compounds (*cf*. *Taraccum ulei*, Maia *et al*. 2013), and anthesis and emission of floral scent does not appear to be synchronised with the nocturnal activity of the beetles (cf. *Annona coriacea*, Gottsberger 1989; *Homalomena propinqua*, Kumano & Yamaoka 2006). Nonetheless, it is plausible that (3*Z*,6*Z*)‐nonadien‐1‐ol and (3*Z*,6*Z*)‐nonadien‐1‐yl acetate in *B. attenuata* function as an aggregation signal for the beetles—advertising feeding and mating opportunities (Allsopp 1990; Gibernau *et al*. 1999; Schiestl 2010; Schiestl & Dötterl 2012). It would be interesting to investigate the possibility that this is a mutualism between plant and beetles, where both partners benefit by maximising opportunity for reproduction (Schiestl & Dötterl 2012).

Our study is the first to demonstrate attraction of pollinating beetles to the scent of Australian Proteaceae. Although Lamont (1982) observed *Pachytricha* sp. visiting and pollinating the strongly scented flowers of *Grevillea leucopteris* Meisn., in this study the plants were cultivated outside of their natural range, and the importance of scent for attraction of the beetles was not demonstrated. Beyond Australia, pollination by diurnally active flower beetles (Scarabaeidae: Cetoniinae) has been documented in several species of *Protea* L. (Proteaceae) in South Africa (Steenhuisen & Johnson 2012). Similar to *B. attenuata*, the beetle‐pollinated *Protea* spp. have fruity, papaya‐like, floral scents and are also visited by nectar‐seeking birds (Steenhuisen *et al*. 2012). However, the two genera differ in that the inflorescences of proteas conform to the ‘painted bowl’ floral syndrome due to presence of large, brightly coloured involucral bracts (Bernhardt 2000; Steenhuisen & Johnson 2012), while the inflorescences of *B. attenuata* resemble more closely the spadices (although lacking the spathe) or the brush‐type inflorescences of Araceae (Bernhardt 2000; Maia *et al*. 2012; Sayers *et al*. 2020). Further, in *Protea* the attraction of diurnal beetles to the flowers appears to be mediated by a combination of visual cues and complex blends of common floral volatiles, including benzaldehyde, β‐linalool, linalool oxide, methyl benzoate and methyl salicylate (Steenhuisen *et al*. 2013). In contrast, in *B. attenuata* attraction of nocturnal beetles appears to be by a relatively simple floral blend, dominated by two compounds otherwise rarely found in flowers– again, similar to beetle‐pollinated Araceae and Annonaceae (Jürgens *et al*. 2000; Maia *et al*. 2012, 2013; Milet‐Pinheiro *et al*. 2017; Pinheiro‐Costa *et al*. 2025). The visual cues are likely also important for attraction of the beetles—consistent with the fact that some beetles were captured also in our control traps (yellow vanes resembling *B. attenuata* inflorescences but without the chemical lure). Nocturnal beetles can be expected to use luminance and colour contrasts to find the flowers even on moonless nights (Gottsberger & Silberbauer‐Gottsberger 1991; Warrant & Dacke 2011), although they likely rely on the visual cues to a lesser extent than diurnally foraging beetles (Balkenius *et al*. 2006).

The function of the pollenkitt suspending the pollen and the short styles of *B. attenuata* merit investigation as potential adaptations to pollination by beetles. In *Banksia*, oily pollen appears to be uniquely associated with *B. attenuata*, with other species having dry, dust‐like pollen (Ladd *et al*. 1996). Close observations of *P. minor* scooping pollen from the pollen presenters with their hairy mandibles suggest that the presence of pollenkitt may facilitate foraging and ingestion of pollen by these beetles, with the lipids in the oil likely providing an additional food reward (Pacini & Hesse 2005; Karolyi *et al*. 2009). Further, the styles in *B. attenuata* are much shorter and less rigid than in *Banksia* species that are pollinated primarily by birds (Wiens *et al*. 1979; Ladd *et al*. 1996). These traits likely facilitate pollination by small animals crawling over the flowers, similar to the spadices of beetle‐pollinated philodendrons (Araceae) (Gibernau *et al*. 1999; Bernhardt 2000; Maia *et al*. 2010, 2023).

While the beetles as a group were the most numerous native visitors, honeyeaters also visited *B. attenuata*. Birds are generally considered to be highly effective pollinators of banksias and many other species in the Proteaceae (e.g. Whelan & Burbidge 1980; Collins & Rebelo 1987; Ashton *et al*. 2025). However, past studies indicate that birds tend to visit *B. attenuata* flowers less frequently than other *Banksia* species, and that they carry comparatively light pollen loads of this species (Hopper 1980; Wooller *et al*. 1983b). Unlike in many other banksias, where stigma‐nectary distances match the bill lengths of the birds that visit the flowers, in *B. attenuata* the styles are too short to consistently transfer pollen to the feathers of most honeyeaters (Wiens *et al*. 1979), including the four species recorded visiting the flowers in the present study. While some pollen may be transferred on the surface of the bill, this mismatch suggests that birds are not the primary pollinators of *B. attenuata*—consistent with the results of Hewes *et al*. (2025), who demonstrated in *Eremophila maculata* (Scrophulariaceae) that honeyeaters with bills that are longer than the stigma‐nectary distance of the flowers are less effective pollinators compared to other species with bills matched to the flowers.

Earlier studies in coastal heathlands found that Honey possums carried pollen of *B. attenuata* (Hopper 1980; Saffer 1998), while evidence from pollinator exclusion experiments suggests they can contribute significantly to pollination of other banksias (Wooller & Wooller 2003; Wawrzyczek *et al*. 2024, 2025a). Numerous records of Honey possums visiting the flowers at heathland sites align with previous studies and suggest that Honey possums can be important pollinators of *B. attenuata* in some habitats. However, the selective pollinator exclusion experiment conducted at one of the woodland sites (this study: supporting information S1) indicated that non‐flying mammals contributed very little to fruit set of *B. attenuata*. This result agreed with the negligible rates of floral visitation detected through camera trapping and direct observation in the woodlands. Interestingly, in an earlier study at the same site, numerous Honey possums were recorded visiting the tree *Banksia menziesii* (Krauss *et al*. 2018; Ashton *et al*. 2025). This discrepancy suggests either a large and rapid change in abundance of Honey possums or that at this site *B. attenuata* is not their preferred food source in summer.

In relation to Hymenoptera, past studies combining quantification of floral visitation with selective exclusion experiments indicate that *A. mellifera* can be effective pollinators of banksias (Vaughton 1992; Gilpin *et al*. 2017; Wawrzyczek *et al*. 2024, 2025a; Ashton *et al*. 2025). However, in the present study, our exclusion experiment could not separate the role of *Apis* in pollination from other insects. Other Hymenoptera that visited the flowers at our study sites, *Amegilla chlorocyanaea* (Apidae) and *Phalerimeris carinifrons* (Scoliidae), likely also contribute to pollination of *B. attenuata* in some populations. However, the three *Amegilla chlorocyanaea* individuals we sampled for pollen all carried mixed pollen loads, meaning that their effectiveness as a pollinator of *B. attenuata* could be diminished by deposition of heterospecific pollen and pollen wastage (Minnaar *et al*. 2019). It would be of interest to test if the diurnal hymenopterans visiting *B. attenuata* are attracted by the same chemical cues as the pollinating scarab beetles, and to compare the per‐visit effectiveness of these two pollinator groups.

Our study contributes to the growing appreciation of the importance of various neglected pollinators, particularly nocturnal invertebrates (Amorim *et al*. 2013; Krug *et al*. 2018; Vlasáková *et al*. 2019; Danaher *et al*. 2020; Blackall *et al*. 2023; Pérez‐Gómez *et al*. 2023; Johnson & Balducci 2024). Indeed, our findings suggest that more attention needs to be directed to the role nocturnal insects play in pollination, in Australia and globally (Macgregor & Scott‐Brown 2020). In the case of *B. attenuata*, the role of nocturnal pollinators was not anticipated due to earlier studies documenting floral visitation and pollen loads for birds and mammals, and similar inflorescence structure to other vertebrate‐pollinated *Banksia* (Wiens *et al*. 1979; Hopper 1980; Whelan & Burbidge 1980; Wooller *et al*. 1983b). As such, our discovery of floral visitation by beetles of *B. attenuata* highlights the importance of conducting nocturnal surveys, particularly for species with unusual floral scents or pollen traits that vary from those of their congeners. Moreover, our discovery of unusual beetle‐attracting compounds in *B. attenuata* shows how investigating floral scent chemistry can deepen our understanding of complex pollination systems and lead toward novel hypotheses for potential mechanisms underlying targeted attraction of pollinators.

## AUTHOR CONTRIBUTIONS

SKW, BB, SLK and RDP conceived and designed the study with support from SEH and RAD. SKW, BB and IMB conducted field and laboratory work with support from SLK, KF and GRF. SKW analysed the data and wrote the manuscript with contribution from all co‐authors.

## FUNDING INFORMATION

This project received funding from the Holsworth Wildlife Research Endowment—Equity Trustees Charitable Foundation & the Ecological Society of Australia awarded to SW, the Australia Pacific Science Foundation (APSF 20049) awarded to SLK and RDP, and a Student Research Award (Ecological Society of Australia) awarded to SKW.

## CONFLICTS OF INTEREST StatemenT

The authors declare they have no conflicts of interest.

## Supporting information


**Fig. S1.** Sampling of floral volatiles and field bioassay using custom‐built vane traps.
**Fig. S2.** Example gas chromatograms.
**Table S1.** Summary of replication for pollinator surveys.
**Table S2.** Volatile compounds detected in the headspace of *Banksia attenuata* flowers.

**Video S1.** Examples of floral visitors and behaviour.

## Data Availability

Floral visitation data is available on GitHub: https://github.com/stanwawrzyczek/Banksia_attenuata_Beetles.
