# Supplementary material for: Attraction of nocturnal scarab beetles by unusual floral volatiles in a Banksia (Proteaceae) with functionally diverse pollinators
Source: Plant Biol (Stuttg). 2026 Jun 2;28(5):1454–66. doi: 10.1111/plb.70226 (PMC13358639; doi:10.1111/plb.70226)
Supplement: Supplementary file 1 — Fig. S1. Sampling of floral volatiles and field bioassay using custom‐built vane traps. Fig. S2. Example gas chromatograms. Table S1. Summary of replication for pollinator surveys. Table S2. Volatile compounds detected in the headspace of Banksia attenuata flowers. [file PLB-28-1454-s002.docx]

SUPPORTING INFORMATION

# Attraction of nocturnal scarab beetles by unusual floral volatiles in a *Banksia* (Proteaceae) with functionally diverse pollinators

STANISLAW K. WAWRZYCZEK; BJÖRN BOHMAN*; SIEGFRIED L. KRAUSS; ISABELLA BUTLER; GAVIN R. FLEMATTI, KEVIN FARNIER, SUSAN E. HOEBEE; ROBERT A. DAVIS, and RYAN D. PHILLIPS

*Corresponding author: [bjorn.bohman@slu.se](mailto:bjorn.bohman@slu.se)

## Contents of this document:

| 1. **Table S1.** Summary of replication for pollinator surveys | page 2 |
| --- | --- |
| 2. Details of floral volatile sampling methods | page 3 |
| 3. Synthesis of (3Z,6Z)-nonadien-1-ol and (3Z,6Z)-nonadien-1-yl acetate | page 4 |
| 4. **Figure S1**. Sampling of floral volatiles and field bioassay using custom-built vane traps | page 5 |
| 5. **Table S2**. Volatile compounds detected in the headspace of *Banksia attenuata* flowers  4. **Figure S2**. Example gas chromatograms | page 6 |
| 6. Selective pollinator exclusion experiment (methods and results) | pages 8-10 |
| 7. References | page 11 |

| **Table S1.** Summary of replication for pollinator surveys for each method at each study site in each flowering season of *Banksia attenuata*. The numbers in the table indicate the number of days during which the surveys were undertaken or the number of cameras used^a^, followed by the total length of the surveys (hrs) in parentheses. | | | | | | |
| --- | --- | --- | --- | --- | --- | --- |
|  | **Site** | **Nov-2020 –Jan-2021** | **Nov-2021 – Jan-2022** | **Nov-2022 – Jan-2023** | **Nov-2023 – Jan-2024** | **Nov-2024 – Jan-2025** |
| Direct observation  D = day  N = night | INR1 | D: 5 (11)  N 4 (6) | |  | D: 1 (1) | D: 1 (1)  N: 1 (1) |
|  | INR2 |  |  |  | D: 1 (1)  N: 1 (1) | D: 2 (2) |
|  | BNP1 |  |  |  | D: 4 (7)  N: 5 (6) | |
|  | BNP2 |  |  | D: 2 (3)  N: 2 (3) | D: 5 (10)  N: 3 (5.5) | |
|  | BNP3 |  |  |  |  | D: 1 (2)  N: 2 (2) |
|  | HV |  |  |  | D: 2 (2)  N: 2 (2) | |
| Day video recordings | INR1 | 9 (15) |  |  | 1 (0.25) | 1 (0.5) |
|  | INR2 |  |  |  | 1 (0.5) | 1 (0.5) |
|  | BNP1 |  |  |  | 3 (2.25) |  |
|  | BNP2 |  |  | 1 (2) | 5 (5) |  |
|  | BNP3 |  |  |  |  |  |
|  | VH |  |  |  | 2 (3h) |  |
| Remote camera trapping | INR1 | 23 (654) | 4 (112) |  |  | 5 (231) |
|  | INR2 |  |  |  |  |  |
|  | BNP1 |  |  |  | 5 (132) |  |
|  | BNP2 |  |  |  | 11 (349) |  |
|  | BNP3 |  |  |  |  | 2 (52) |
|  | HV |  |  | 2 (33) | 10 (356) |  |
| ^a^ We avoided surveying multiple inflorescences on the same plant simultaneously, however, occasionally the same plant was re-surveyed at a different time. | | | | | | |

## Details of floral volatile sampling and chemical analyses

To determine the composition of *B. attenuata* floral scent, floral volatiles were sampled in the laboratory from the headspace of freshly cut flowers using Solid Phase Microextraction (SPME) fibres (DVB/CAR/PDMS fibres, Supelco, Sigma-Aldrich, Germany) and analysed by gas chromatography-mass spectrometry (GC-MS). Five inflorescences were collected from INR sites and one from the nearby Yeal Nature Reserve. The inflorescences were transported to the laboratory and processed within 4 hrs of collection. Pruned branches bearing the inflorescences (with some leaves attached) were placed in glass beakers and covered tightly with aluminium foil (Figure S1 A). The beakers with the flowers were placed in a closed cabinet and volatiles were sampled for approximately 12 hrs, overnight. To the human senses, the quality of the floral scent was not affected by cutting the branches off the trees, and the inflorescences remained strongly fragrant at the end of the sampling period. An additional sample was collected from leaves without the flowers to identify non-floral volatiles. A negative control sample was collected from ambient air to identify potential contaminants. Within 1 hour of sampling, the fibres were inserted into a gas chromatograph (HP 6890, Hewlett-Packard, USA) coupled with a mass spectrometer (HP 5973, Hewlett-Packard, USA) with UHP helium as carrier gas (flow rate of 0.7 mL min^-1^). Volatiles were desorbed from the fibres for 1 min at 280° C and transferred in a splitless mode (purge time: 1 min) to a nonpolar column (DB-5ms, 50 m x 0.2 mm x 0.33 µm, Agilent, USA). The oven was heated at a rate of 5° C min^-1^ from 40 ° C to 200° C and then at 10° C min^-1^ up to 280° C min^-1^ and held for 5 min.

To allow determination of compounds by co-injection with authentic standards, two additional samples of floral volatiles were collected using dynamic headspace sampling with Tenax filters (50 mg, Tenax® TA, 60-80 mesh, Supelco, Sigma-Aldrich). Fresh *B. attenuata* inflorescences were placed in 1 L clear polycarbonate jars (Nalgene®, Sigma-Aldrich) with fitted lids. Using a vacuum pump, air (0.5 L min^-1^) was pulled through a gas washing bottle, Teflon tubing, sampling jars, and activated (N_2_ gas, 250 °C, 60 min) Tenax® filters (Figure S1 B). The samples were collected over 24 hours and eluted from the traps with 25 µL of 1:1 (v/v) hexene:acetone. An aliquot (1 µL) of each sample was injected into the GC-MS and processed by the same method as the SPME fibres described above.

GC-MS data was analysed with AMDIS (version 2.73) using automated deconvolution. All compounds that were detected above an abundance threshold of 1000 (clearly visible, distinct peaks) were tentatively identified by manually comparing mass spectra and retention indices (calculated for the DB-5 column relative to C8-20 alkane series) with the best-matching compounds from the NIST20 mass spectral library using NIST MS Search (version 2.3, NIST, USA). Peaks for which no match could be found in the library were left as unidentified. The three most abundant compounds that were consistently detected through both SPME and dynamic headspace sampling were identified by co-injection with reference compounds on two columns: one non-polar (details as above) and one polar (AT-WAXms, 30 m × 0.25 mm × 0.25 µm, Grace-Davison, Australia), using a uniform, slower temperature gradient of 4° C min^-1^ from 40 ° C to 280° C.

Preliminary tests were conducted to check if the composition of *B. attenuata* floral scent varied consistently between day and night and to determine the optimal length of SPME sampling. Briefly, floral volatiles were collected using SPME from three inflorescences over 3.5 hrs in the afternoon with the flowers exposed to indirect sunlight. The samples were analysed using GC-MS and the same inflorescences were sampled with the same fibres for 3.5 hrs in the evening, with the flowers placed in a dark cabinet. The comparison of the GC-MS data revealed some variation in the relative amounts of some compounds between day and night samples and between fibres, however, no consistent pattern emerged and overall emissions were approximately equal. In a separate comparison, six SPME fibres were used to sample volatiles from a single inflorescence. Three of the fibres were analysed after 3 hrs and the other three after 13 hrs. Comparison of GC-MS data revealed that after 13 hours the total amount of compounds was higher, the less-volatile compounds were detected more reliably, and there was less variation among the fibres. Overall, these comparisons indicated that the best approach was to sample over a longer period of time to maximise detection of compounds.

## Synthesis of (3*Z*,6*Z*)-nonadien-1-ol and (3*Z*,6*Z*)-nonadien-1-yl acetate

(3*Z*,6*Z*)-Nonadien-1-ol was synthesised by coupling of 3-butyn-1-ol and 1-bromo-2-pentyne, followed by a highly *cis*-selective Lindlar-catalysed hydrogenation (Kerdesky et al. 1987). (3*Z*,6*Z*)-Nonadien-1-yl acetate was formed from the alcohol via a lipase-mediated transesterification with vinyl acetate employing Amano PS lipase from *Pseudomonas cepacia* (Bohman & Unelius 2009). Both compounds were prepared in > 97 % purity, with spectroscopic data (^1^H NMR, ^13^C NMR and GC-MS) in agreement with the literature (Sasaki *et al.* 2011).


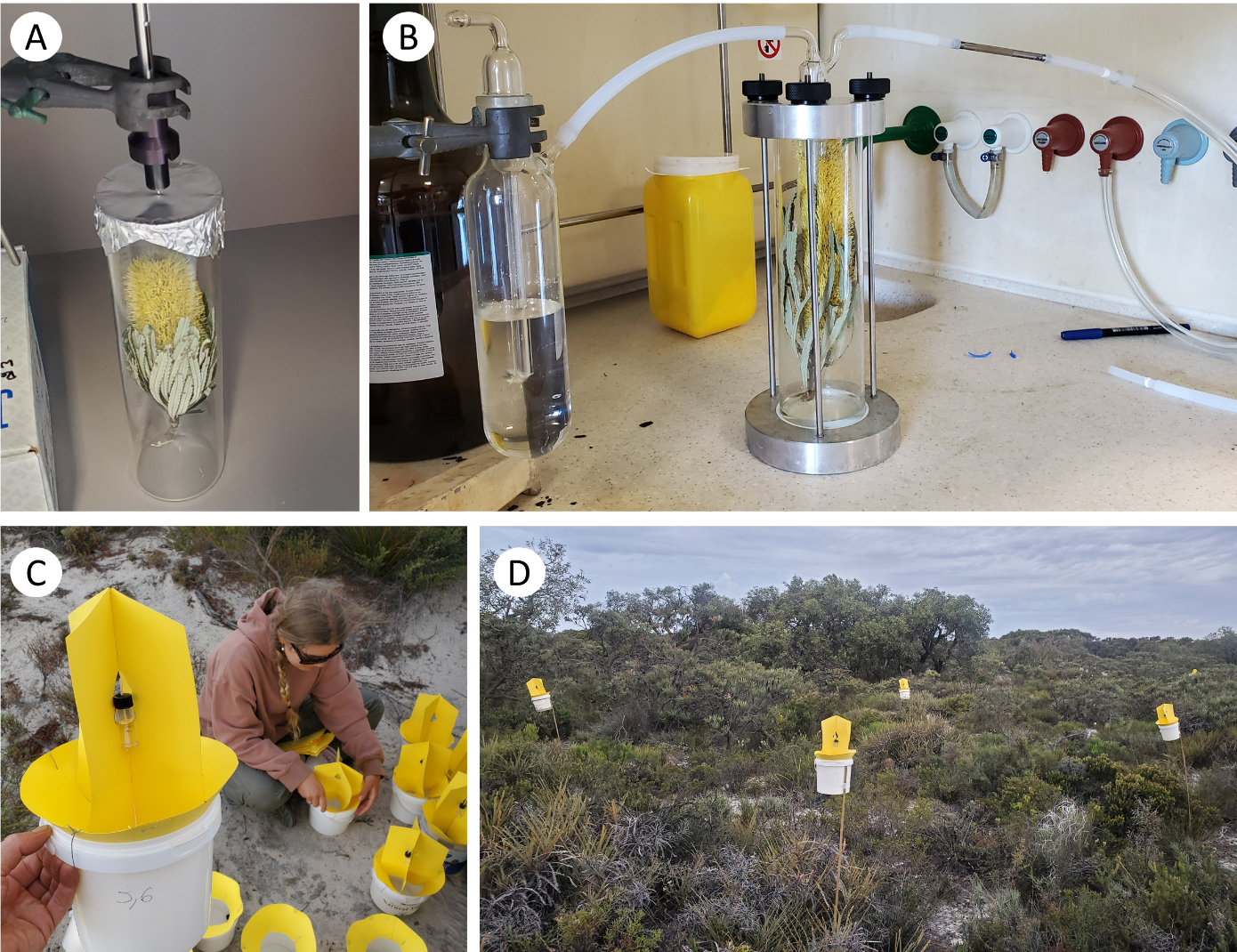


**Figure S1**. (A) SPME sampling of *Banksia attenuata* inflorescence, (B), dynamic sampling with Tenax ® traps, (C) custom vane traps being assembled in the field, showing a 4 ml vial containing the lure fitted with a cotton wick within a Teflon ® tube attached to side of the cross panel, and (D) traps set up among flowering plants of *Banksia attenuata* at Badgingarra National Park (BNP2). Two of the four traps in each array like the one shown were baited with the synthetic lure comprising 0.5 mg of 3,6-nonadien-1-yl acetate and 0.5 mg of 3,6-nonadien-1-ol per 1 mL of hexane.

| **Table S2.** Relative abundance of the volatile compounds detected using SPME in the headspace of *Banksia attenuata* flowers and leaves. Values in the table are proportion of total ion current after square root transformation in each sample of *B. attenuata* flowers with leaves attached (F+L) and leaves only (L); calculated mean proportion in F+L. TIC = total peak area, excluding known contaminants. RT = retention time (min); RI = Kovats retention index calculated for DB-5 semi-standard non-polar column, based on C8-20 series. In **bold**, the three most abundant compounds that were confirmed with authentic standards. Apart from these three, the compounds were tentatively identified by comparison of RI and MS spectra with NIST20 library. Abundance values <0.0001, indicating compounds were not detected at the specified threshold, were omitted from the table for clarity. m/z = ion mass to charge ratio of the main ions detected in GC peaks that did not match entries in NIST20 library (in descending order of area under the peak). | | | | | | | | | | | | | | | | | | |  |
| --- | --- | --- | --- | --- | --- | --- | --- | --- | --- | --- | --- | --- | --- | --- | --- | --- | --- | --- | --- |
| **Compound** | **RT** | **RI** | **F+L 1** | **F+L 2** | | | **F+L 3** | | **F+L 4** | | **F+L 5** | | **F+L 6** | | **L 6** | **Mean (F+L)** | | | |
| β-Myrcene | 16.48 | 991 | 0.0345 |  | | | 0.0106 | |  | | 0.0175 | | 0.0112 | |  | 0.012 | | | |
| 3-Hexene-1-yl acetate | 16.91 | 1004 | 0.0416 | 0.0184 | | | 0.0389 | | 0.0824 | |  | |  | |  | 0.030 | | | |
| 1-Hexyl acetate | 16.91 | 1005 |  |  | | |  | | 0.0086 | |  | |  | |  | 0.001 | | | |
| Unidentified monoterpene (m/z: 91, 119, 134, 77, 92, 105) | 17.22 | 1015 | 0.0198 |  | | | 0.0202 | |  | | 0.0171 | | 0.0080 | |  | 0.011 | | | |
| 2-Ethyl-hexan-1-ol | 17.28 | 1016 |  |  | | |  | |  | |  | |  | | 0.0226 |  | | | |
| **(*E*)-β-Ocimene** | **18.41** | **1055** | **0.5312** | **0.0160** | | | **0.2561** | | **0.0456** | | **0.3494** | | **0.3535** | | **0.5707** | **0.259** | | | |
| Homomenthol | 18.42 | 1052 |  |  | | |  | |  | |  | |  | | 0.0242 |  | | | |
| Linalool | 20.09 | 1105 |  |  | | |  | |  | |  | | 0.1761 | |  | 0.029 | | | |
| p-(1-propenyl)-toluene | 20.01 | 1102 |  |  | | | 0.0167 | |  | |  | |  | |  | 0.003 | | | |
| Unidentified monoterpenoid (m/z: 136, 67, 76, 91) | 20.15 | 1106 |  |  | | | 0.0419 | |  | | 0.0107 | |  | |  | 0.009 | | | |
| Alloocimene isomer 1 | 21.01 | 1134 | 0.0636 |  | | | 0.0270 | |  | | 0.0350 | | 0.0460 | | 0.0214 | 0.029 | | | |
| Cosmene | 21.17 | 1139 | 0.0308 |  | | | 0.0212 | |  | | 0.0259 | | 0.0229 | | 0.0119 | 0.017 | | | |
| Alloocimene isomer 2 | 21.43 | 1147 | 0.0574 |  | | | 0.0269 | |  | | 0.0246 | | 0.0410 | | 0.0213 | 0.025 | | | |
| 2,6-Nonadienal | 21.82 | 1159 | 0.0353 |  | | | 0.0271 | | 0.0381 | | 0.0314 | | 0.1307 | |  | 0.044 | | | |
| **(3*Z*,6*Z*)-Nonadien-1-ol** | **21.95** | **1162** | **0.1184** | **0.2977** | | | **0.1265** | | **0.2861** | | **0.1082** | | **0.0164** | |  | **0.159** | | | |
| Ethyl benzoate | 22.52 | 1181 |  |  | | | 0.0452 | |  | |  | |  | |  | 0.008 | | | |
| (*E*)-Phenylacetaldehyde O-methyloxime ^a^ | 22.61 | 1184 |  |  | | | 0.0212 | |  | | 0.0414 | |  | |  | 0.010 | | | |
| (*Z*)-Phenylacetaldehyde O-methyloxime^a^ | 22.79 | 1190 |  |  | | | 0.0187 | |  | | 0.0380 | |  | |  | 0.009 | | | |
| Methyl salicylate | 23.31 | 1206 | 0.0346 | 0.0257 | | | 0.0127 | |  | | 0.0820 | |  | |  | 0.026 | | | |
| **(3*Z*,6*Z*)-Nonadien-1-yl-acetate** | **25.94** | **1296** | **0.0330** | **0.5600** | | | **0.2762** | | **0.4983** | | **0.0417** | | **0.1619** | |  | **0.262** | | | |
| 2,6-Nonadienyl acetate | 26.34 | 1313 |  | 0.0204 | | |  | | 0.0173 | |  | |  | |  | 0.006 | | | |
| Methylbicyclo[3.2.1]octa-2,6-diene | 29.46 | 1423 |  | 0.0171 | | |  | | 0.0185 | |  | |  | |  | 0.006 | | | |
| Caryophyllene | 29.58 | 1432 |  |  | | |  | |  | |  | |  | | 0.1296 |  | | | |
| Gymnomitrene | 31.01 | 1490 |  | 0.0143 | | |  | |  | | 0.0689 | |  | |  | 0.014 | | | |
| Methyl isoeugenol | 31.54 | 1512 |  | 0.0121 | | | 0.0051 | |  | |  | |  | |  | 0.003 | | | |
| α-Farnesene | 31.30 | 1502 |  | 0.0120 | | | 0.0028 | |  | | 0.0479 | | 0.0318 | | 0.1308 | 0.016 | | | |
| (*E*)-Nerolidol | 33.01 | 1571 |  | 0.0063 | | | 0.0050 | | 0.0053 | |  | | 0.0005 | |  | 0.003 | | | |
| Pentadecanal | 35.94 | 1690 |  |  | | |  | |  | | 0.0334 | |  | |  | 0.006 | | | |
| 2-Ethylhexyl salicylate | 37.35 | 1810 |  |  | | |  | |  | | 0.0269 | |  | | 0.0675 | 0.004 | | | |
| TIC |  |  | 318545 | | 259214 | 433932 | | 321306 | | 361004 | | 810996 | | 229466 | | |  |  |  |

^a^ Retention order based on results of Delarot & Jaquier (2009) who injected standard compounds on a similar DB-1 column.

##
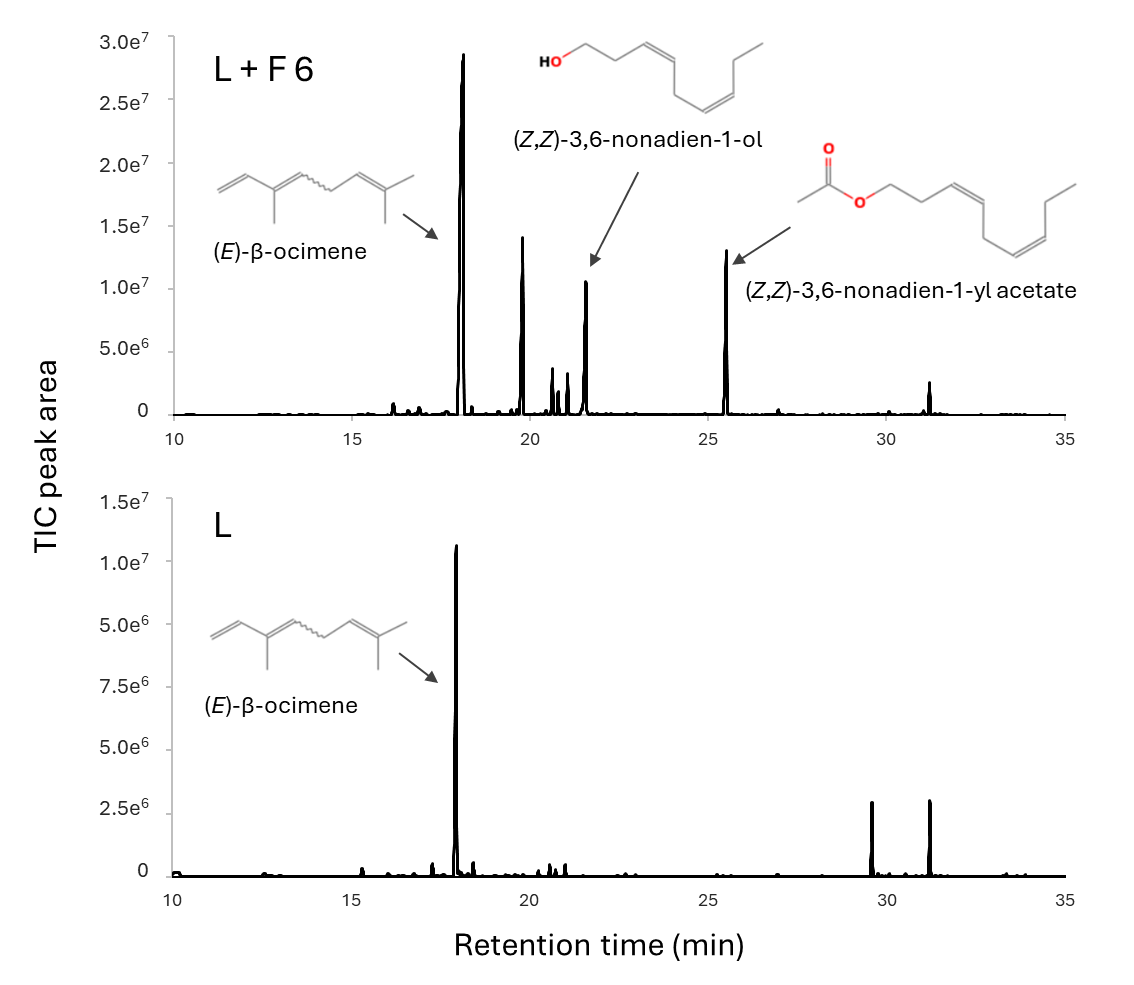


**Figure S2.** Example chromatograms comparing samples collected from the headspace of *B. attenuata* inflorescences (with leaves attached, L + F 6 sample) and leaves without flowers (L 6 sample, taken from the same plant) indicating that (*E*,*Z*)-3,6-nonadien-1-ol and (*E*,*Z*)-3,6-nonadien-1-yl acetate were a major component of the floral scent and were not detected in the leaves, while (*E*)-β-ocimene was a major component also of the leaf volatiles (compound structures credit: NIST Chemistry WebBook).

## Selective pollinator exclusion experiment

### Methods

This experiment was conducted between 23-Nov-2020 and 20-Jan-2021 at Ioppolo Nature Reserve (INR1). Before anthesis, 85 inflorescences on 39 trees were assigned to one of the following pollination treatments: (1) autogamy: all pollinators excluded by a wire cage covered with fine mesh (0.1 x 0.1 cm opening); (2) small insect pollination: birds, mammals and large insects excluded from the flowers by either UV-stable black nylon mesh nets supported on a steel frame to cover the entire young tree or a rigid wire cage over an individual inflorescence secured to the branch with zip ties (1.5 x 1.5 cm opening); (3) non-flying mammal pollination: flying pollinators excluded by fine mesh nets either (i) supported on a steel frames to cover entire young trees except for a 4 cm gap at the bottom or (ii) a rigid cage covered with fine mesh over individual inflorescences, as in 1 but leaving the bottom of the cage open to allow scansorial mammals to access the flowers; (4) open pollinated control: unmanipulated inflorescences marked at the start of anthesis. See supporting information for photos of the structures in the field (Figure S1) and for full details of replication (Table S2).

| **Table S3.** Details of replication for the selective pollinator exclusion experiment with *Banksia attenuata* (inflorescences / plants). | | | | |
| --- | --- | --- | --- | --- |
|  | **Autogamy**  **(all pollinators excluded)** | **Non-flying mammals only (flying pollinators excluded)** | **Small insects only (large-bodied pollinators excluded)** | **Open pollinated control** |
| ***N* (all)** | 6 / 6 | 29 / 23 | 28 / 21 | 22 / 18 |
| ***N* (individual inflorescence exclusions)** | 6 / 6 | 19 / 19 | 16 / 16 | 22 / 18 |
| ***N* (whole-tree exclusions)** | 0 | 10 / 4 | 12/ 5 | 0 |

Where possible, all treatments were replicated on each tree. However, this was not feasible for the whole-tree exclusions, which comprised 23 inflorescences across 9 plants. The efficacy of the exclusion treatments was confirmed by camera traps and direct observation, with only very small numbers of *A. mellifera* found breaching the whole-plant non-flying mammal pollination treatment. The experimental inflorescences were collected on 21-Dec-2021 and fruit set was scored in the laboratory.

To compare fruit set in different pollination treatments we used ‘glmmTMB’ (Brooks *et al.*, 2017) in R version 4.2.3 (R Core Team, 2021) to fit negative binomial distribution (nbinom2) to the data with a log-link function. We set maternal plant as random effect in the model. Pairwise differences among pollination treatment groups were assessed through estimation of marginal means using ‘emmeans’ (Lenth, 2025).


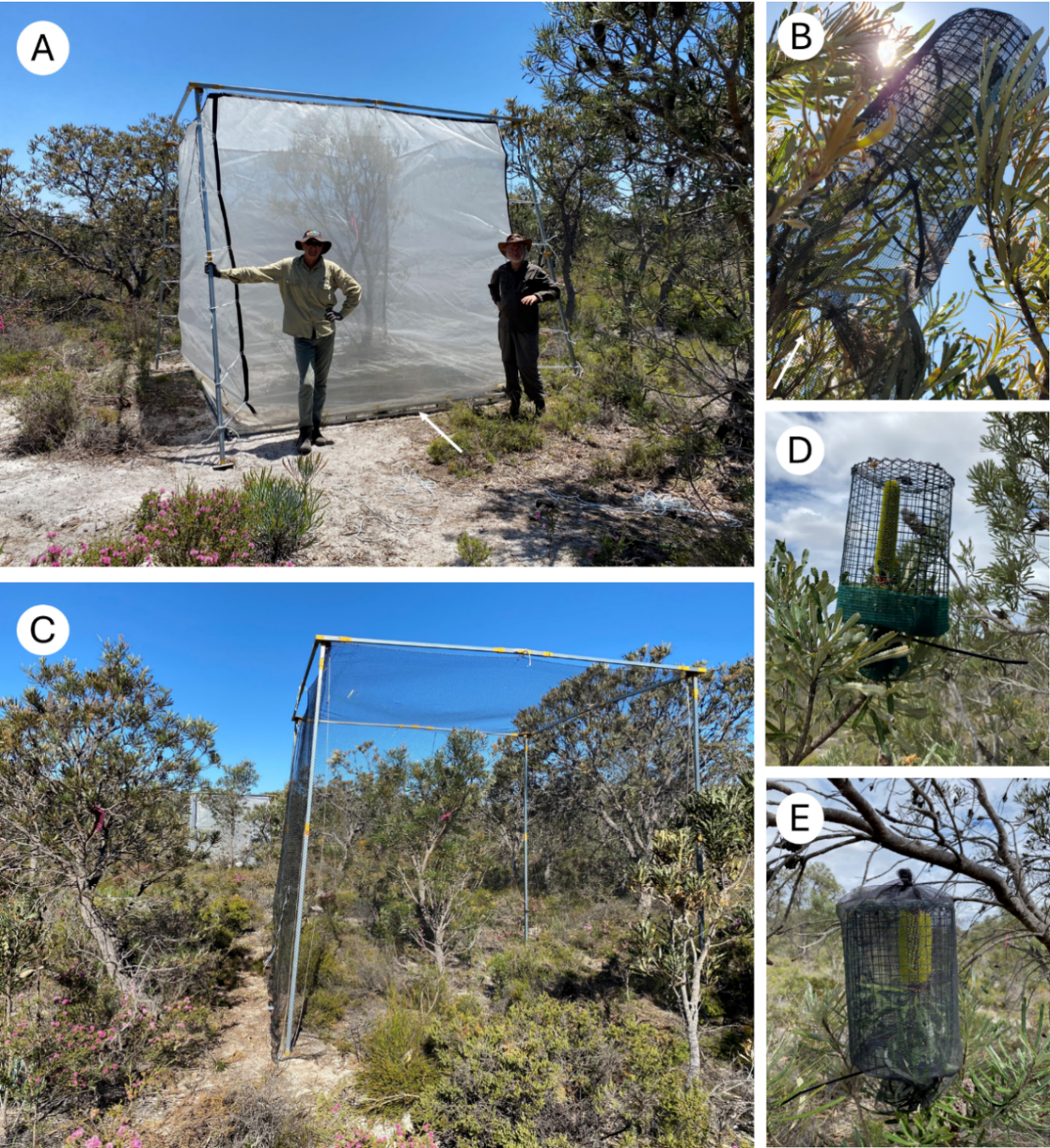


**Figure S3.** Examples of pollinator exclusion cages over *Banksia attenuata* plants in the field at Ioppolo Nature Reserve (INR1). (A-B), whole-tree and single inflorescence mammals-only treatment (flying pollinators excluded by fine mesh net, small mammals can access the flowers through the opening at the bottom, arrowed); (C-D), Whole tree and single inflorescence small insects-only treatment (vertebrates and large insects excluded by coarse wire mesh cage 1 cm^2^ opening); (E), autogamy treatment (all pollinators excluded by fine mesh closed tightly over the stem below the inflorescence).

### Results

Pollinator surveys concurrent with this experiment at INR detected no honey possums and only sporadic visitation by Brown honeyeaters, while large invertebrates (that would also be excluded by the lattice cages/nets) comprised *Pachytricha minor* and *Dasypodia selenophora*. The small insects visiting the flowers were *Pachytricha occidentalis* and *Apis mellifera*.

Inflorescences from which all pollinators were excluded did not produce any fruit (*N* = 6). Fruit set was very low when only mammals were allowed to access the flowers, with most inflorescences failing to produce any fruit (mean = 0.7 ± 0.5 SE follicles per cone, *N* = 29). Fruit set increased substantially when only small insects could access the flowers (mean = 6.4 ± 1.5 SE follicles per cone, *N* = 28) but was still considerably lower than in the open pollinated control (mean = 11.2 ± 1.9 SE, *N* = 22; Figure S3).Pairwise tests based on the GLMM (Table S4) indicated strong evidence that fruit set was reduced when all flying pollinators were excluded and only mammals could access the flowers (‘Mammals’ treatment vs ‘Open’ control, *P* < 0.001). However, because of the high variance in the data, there was no evidence that fruit set was reduced when only small insects could access the flowers (‘Small insects’ treatment vs ‘Open’ control, *P* = 0.26; Figure S3).


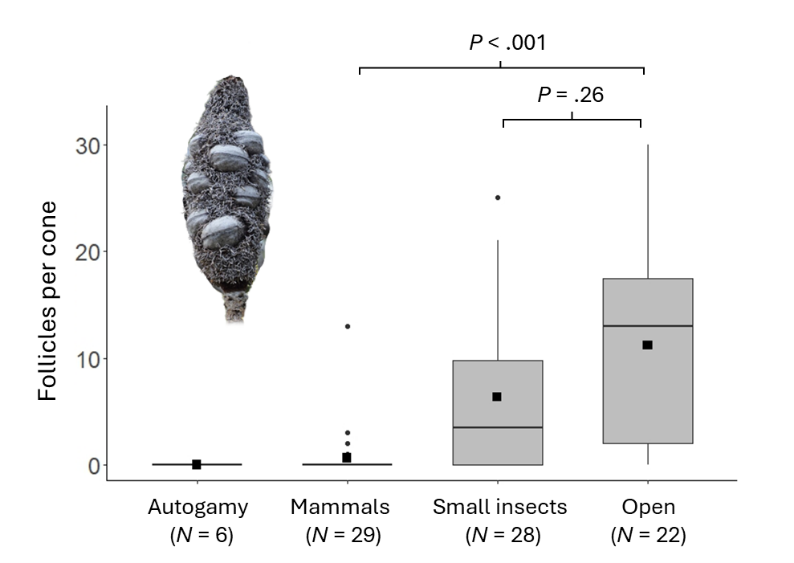


**Figure S4**. Fruit set in the selective pollinator exclusion experiment with *Banksia attenuata*. Black squares indicate group means (*N* = number of inflorescences in each group). *P*-values for pairwise comparisons estimated using ‘emmeans’ based on GLMM (Table S4). The image shows a *B. attenuata* infructescence with mature woody follicles.

| **Table S4.** Results of the GLMM fitting negative binomial comparing fruit set following selective pollinator exclusion if *Banksia attenuata* (*N* = 79 (38) inflorescences (trees)). Autogamy treatment was not included in the comparison because no fruit were produced. | | | | |
| --- | --- | --- | --- | --- |
| **Treatment** | **Estimate** | **SE** | **z** | **P** |
| **intercept** | -0.72 | 0.54 | -1.35 | 0.17 |
| **Small insects** | 2.59 | 0.63 | 4.08 | < 0.001 |
| **Open (control)** | 3.14 | 0.64 | 4.88 | < 0.001 |
|  |  |  |  |  |

## References:

Bohman, B.,Unelius C. R. (2009). Synthesis of all four stereoisomers of 5-hydroxy-4-methyl-3-heptanone using plants and oyster mushrooms. *Tetrahedron*, **65**, 8697-8701.

Brooks M.E., Kristensen K., van Benthem K.J., Magnusson A., Berg C.W., Nielsen A., Skaug H.J., Mächler M., Bolker B.M. (2017) glmmTMB balances speed and flexibility among packages for zero-inflated generalized linear mixed modeling. *R Journal*, **9,** 378–400.

Delort, E., Jaquier, A. (2009). Novel terpenyl esters from Australian finger lime (*Citrus australasica*) peel extract. *Flavour and fragrance journal*, **24**, 123-132.

Kerdesky, F. A., Schmidt, S. P., Holms, J. H., Dyer, R. D., Carter, G. W., Brooks, D. W. (1987). Synthesis and 5-lipoxygenase inhibitory activity of 5-hydroperoxy-6, 8, 11, 14-eicosatetraenoic acid analogs. *Journal of Medicinal Chemistry*, **30**, 1177-1186.

Lenth R. (2025) emmeans: Estimated Marginal Means, aka Least-Squares Means. R package version 1.11.2-8

Sasaki, K., Urabe, D., Arai, H., Arita, M., Inoue, M. (2011). Total synthesis and bioactivities of two proposed structures of maresin. *Chemistry–An Asian Journal*, **6**, 534-543.
